# Molecular Players Involved in the Interaction Between Beneficial Bacteria and the Immune System

**DOI:** 10.3389/fmicb.2015.01285

**Published:** 2015-11-18

**Authors:** Arancha Hevia, Susana Delgado, Borja Sánchez, Abelardo Margolles

**Affiliations:** Instituto de Productos Lácteos de Asturias, Consejo Superior de Investigaciones CientíficasVillaviciosa, Spain

**Keywords:** *Bifidobacterium*, immunomodulation, *Lactobacillus*, molecular players, probiotic

## Abstract

The human gastrointestinal tract is a very complex ecosystem, in which there is a continuous interaction between nutrients, host cells, and microorganisms. The gut microbiota comprises trillions of microbes that have been selected during evolution on the basis of their functionality and capacity to survive in, and adapt to, the intestinal environment. Host bacteria and our immune system constantly sense and react to one another. In this regard, commensal microbes contribute to gut homeostasis, whereas the necessary responses are triggered against enteropathogens. Some representatives of our gut microbiota have beneficial effects on human health. Some of the most important roles of these microbes are to help to maintain the integrity of the mucosal barrier, to provide nutrients such as vitamins, or to protect against pathogens. In addition, the interaction between commensal microbiota and the mucosal immune system is crucial for proper immune function. This process is mainly performed via the pattern recognition receptors of epithelial cells, such as Toll-like or Nod-like receptors, which are able to recognize the molecular effectors that are produced by intestinal microbes. These effectors mediate processes that can ameliorate certain inflammatory gut disorders, discriminate between beneficial and pathogenic bacteria, or increase the number of immune cells or their pattern recognition receptors (PRRs). This review intends to summarize the molecular players produced by probiotic bacteria, notably *Lactobacillus* and *Bifidobacterium* strains, but also other very promising potential probiotics, which affect the human immune system.

## The human gut microbiota and the immune system

From the early stages of life, one of the most important roles of the gut microbiota is to contribute to the development of a proper immune system. Normally, humans live in a homeostatic symbiosis with their gastrointestinal microbes, providing them with nutrients and a friendly environment, whereas the microbiota aids in the appropriate development and maintenance of the host's gut mucosa. Epithelial function is influenced by direct host/microbiota interactions and microbial metabolism. The large intestine acts as an anaerobic bioreactor for the enteric bacterial community, which is fueled by host diet components that cannot be processed in the small intestine, as well as by endogenous nutrients, such as host glycans from mucus and cell debris released from epithelial cells. Additionally, the microbiota synthesizes essential amino acids, vitamins, and short chain fatty acids (SCFAs) by degrading a variety of proteins and otherwise non-digestible polysaccharides (Sekirov et al., 2010).

**Figure 1:**
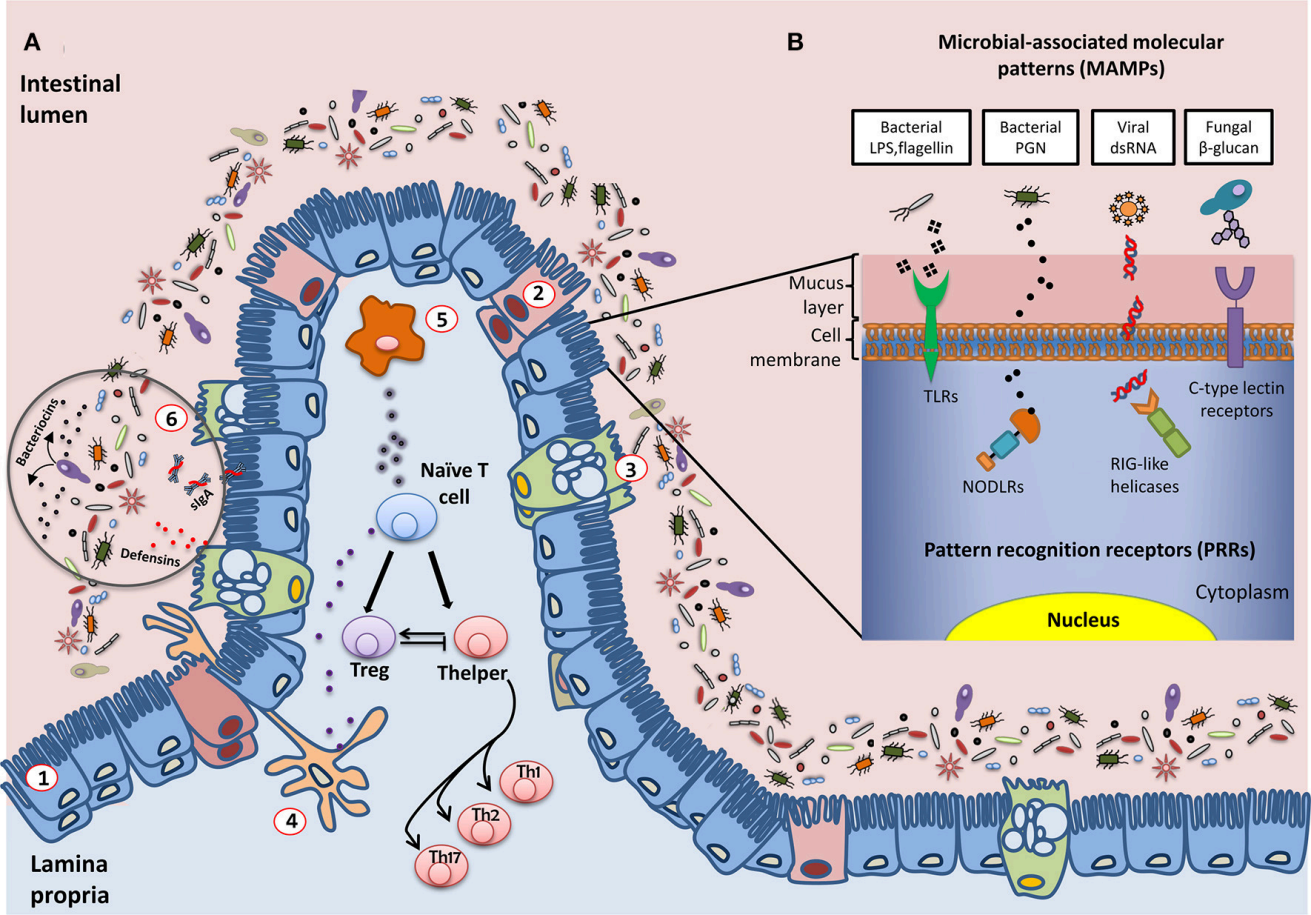
**Schematic representation of the interactions established between the intestinal microbiota and the host immune system**. **(A)** General overview of the epithelium in contact with multiple species of microorganisms that constitute the intestinal microbiota: (1) enterocytes; (2) M cells; (3) Goblet cells; antigen presentation cells (APC): (4) dendritic cells and (5) macrophages; (6) defensins, bacteriocins, and secreted IgA (sIgA) also play an important role in controlling the levels of the different populations of microorganisms. A fine-tuned balance of Tcell maturation toward Treg or Thelper cells must be established to assure the tolerogenic response of the host immune system. **(B)** Examples of molecular interactions between microbial antigens and host cells through Pattern Recognition Receptors (PRRs). LPS, lipopolysaccharide; PGN, peptidoglycan; dsRNA, double-strand RNA; TLR, toll-like receptor; NODLR, nucleotide-binding oligomerization domain-like receptors; RIG-like helicases, retinoic acid-inducible gene 1 like helicases.

The commensal microbiota ensures the mechanical integrity of the mucosal barrier, thereby offering protection against harmful pathogenic microbes (Figure 1). Commensal bacteria can adhere to the intestinal mucus and competitively inhibit the adhesion of enteropathogens; they also produce bacteriocins and SCFAs, compounds that are able to inhibit the growth of other microorganisms. Additionally, some antimicrobial metabolites, such as the defensins secreted in the intestine, contribute to the host's control of these microorganisms (Salzman et al., 2007). Further protection of the host is provided by inducing the mucosal immune system to produce immunoglobulin A, which is released in the intestinal lumen in large amounts and limits local bacterial colonization, thereby preventing bacteria from penetrating the epithelium (Salzman et al., 2007).

A major issue is how the intestine distinguishes between the abundant, normal microbiota and rare pathogens. The immune system fights pathogenic bacteria, but tolerates the presence of commensal species, even though their cellular structures are quite similar and they have common mechanisms of interacting with host immune cell receptors; this phenomenon is called immune tolerance. In this way, our immune cells differentiate between commensals and pathogens. This is carried out by our innate immune system through pattern recognition receptors (PRRs) (Figure 1), including Toll-like receptors (TLRs), transmembrane receptors that scan the external milieu of the intestinal lumen, and Nod-like intracellular receptors (NODLR), which guard the cytoplasmic space (Claes et al., 2015; Sellge and Kufer, 2015). Other PRRs have also been described, such as C-type lectin receptors, formylated peptide receptors, retinoic acid-inducible (RIG)-like helicases, and intracellular interleukin-1 (IL-1)-converting enzyme protease-activating factor (Denes et al., 2012; Bufe et al., 2015; Dambuza and Brown, 2015; Yao et al., 2015). PRRs are able to specifically recognize and bind different microbial macromolecular ligands, which are designated as microbial-associated molecular patterns (MAMPs), such as lipopolysaccharide, flagellin and other proteins, bacterial peptidoglycan, viral RNAs, and fungal carbohydrates. As a result, the T cell subset involved in regulating the immune balance is finely tuned by the host and the microbes with which it interacts, and disequilibrium between effector T helper (Th) and regulatory T cells (Treg) leads to impaired immune responses (Noack and Miossec, 2014; Nyirenda et al., 2015; Yousefi et al., 2015). Effector Th cells are derived from progenitor naïve CD4+ T cells via maturational processes that are induced by antigenic stimulation. Their function depends on complex interactions with antigen-presenting cells (APCs) in a permissive environment, which is characterized by the antigen type and load, costimulatory molecules, and cytokine signaling. CD4+ T cells may differentiate into different Th phenotypes (mainly Th1, Th2, and Th17) that produce distinct cytokines with different biological functions, or they may evolve into the inducible Treg lineage, which performs immunomodulatory functions (Sakaguchi et al., 2010; Wing and Sakaguchi, 2014). The Th1 subgroup recognizes intracellular pathogens and mainly produces IL-2, interferon (IFN), and tumor necrosis factor alpha (TNFα), thereby supporting typical cellular immunity. Th2 cells, which are essential for eliminating extracellular pathogens such as helminths, express IL-4, IL-5, IL-10, and IL-13, which aid humoral immunity. The Th17 subset, which is involved in fighting Gram-negative bacteria, fungi, and some protozoa, secretes IL-17, which has strong pro-inflammatory effects. Overall, Th responses are accurately balanced to avoid both self-antigen reactivity and excessive reactions to antigens. In fact, dysregulated Th1 responses drive cell-mediated autoimmune disorders, and enhanced Th2 activity is involved in atopy, whereas Th17 cells are probably responsible for chronic tissue inflammation. In contrast, skewing the response away from Treg cells may lead to the onset and/or progression of autoimmune diseases in humans (Eisenstein and Williams, 2009).

## Probiotics and the immune system

During the last few years, it has been proposed that the intestinal microbiota can be positively modulated by the administration of bacteria or bacterial substrates, and it is likely that, to some extent, this might lead to a significant modulation of the immune system (Dongarrà et al., 2013; Sánchez et al., 2015; Scott et al., 2015). To this end, substantial research efforts are concentrated on using probiotics as potential modulators of gut microbial community. Probiotics are commensal microorganisms that are present in the intestinal tract and in many fermented foods, and they are defined as “live microorganisms that, when administered in adequate amounts, confer a health benefit on the host” (Hill et al., 2014). The vast majority of probiotic bacteria are Gram-positive strains, mainly species of the *Lactobacillus* and *Bifidobacterium* genera, although some non-pathogenic strains of *Escherichia coli* and certain yeasts are also considered to be probiotics. Currently, there is an increasing interest in considering some common colonizers of the human gut to be novel probiotics, because of their potential health properties; they are called *emerging probiotics* (Hill et al., 2014; Rodríguez et al., 2015). Some examples are *Faecalibacterium prausnitzii* and *Akkermansia muciniphila*.

**Figure 2:**
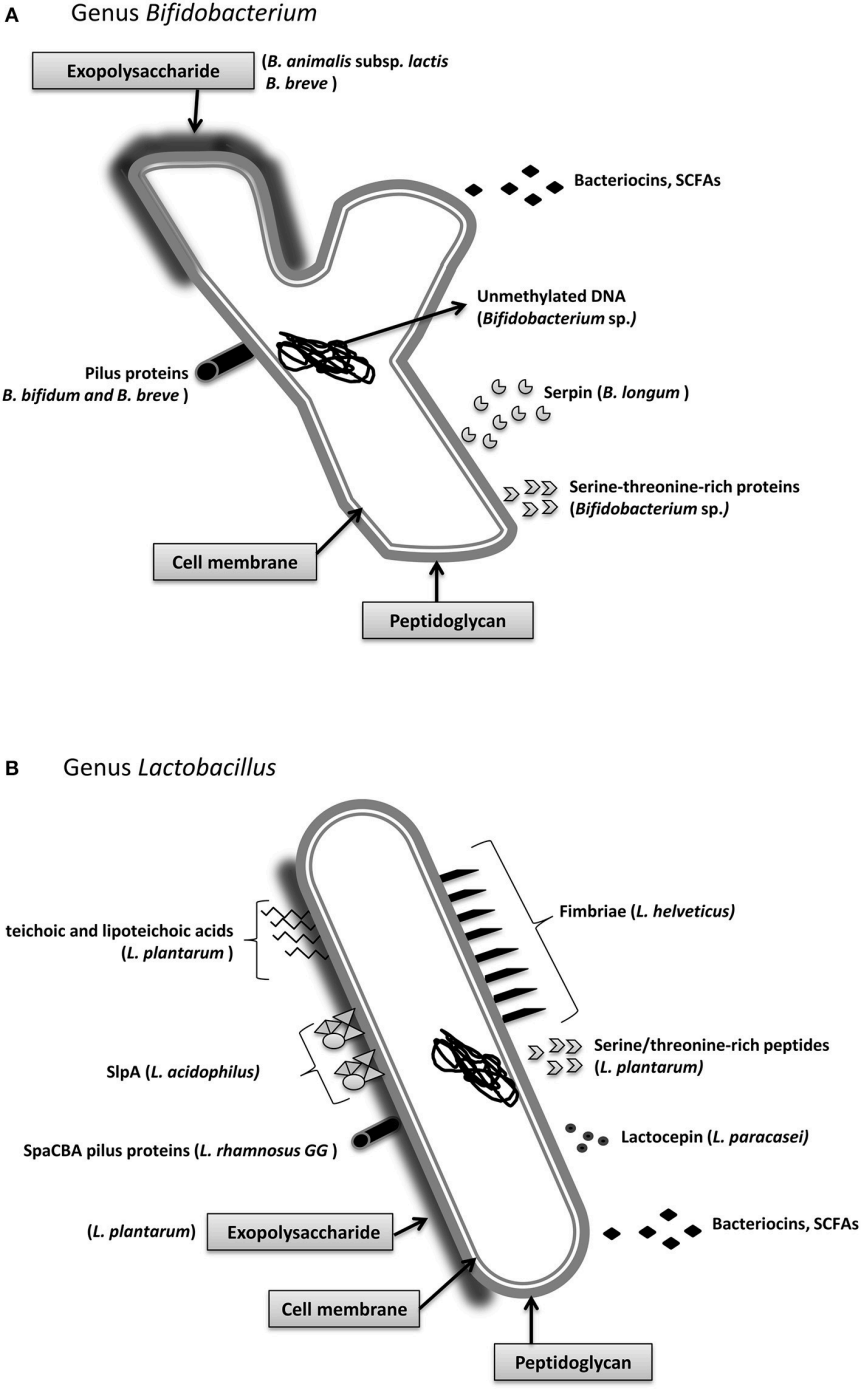
**Main molecular effectors that are able to trigger immunomodulatory responses in the host: ***Bifidobacterium*** (A) and ***Lactobacillus*** (B)**. Some of these effectors are species-specific, such as the S-layer protein A from *Lactobacillus acidophilus*, whereas others, such as short chain fatty acids, are secreted by the vast majority of strains. Detailed information about the mechanisms and the molecular effectors is included in Section Immunomodulatory Effectors.

Probiotics can exert their beneficial properties in a wide range of ways, including direct cell-to-cell contact in the human gut, by secreting diverse molecules that act as the final mediators of probiotic crosstalk, or through cross-feeding mechanisms. The chemical composition of the molecular effectors is very diverse and consists of proteins that are secreted into the extracellular milieu or localized on the surface of the bacteria, low molecular weight peptides, amino acids, cell-wall polysaccharides or components, bacterial DNA, or SCFAs (Macpherson and Harris, 2004; Turroni et al., 2013). Given the different molecular natures of these molecular effectors, their mechanisms of action are very diverse. Therefore, this review includes only a summary of the molecular bases underlying the immunomodulatory properties of probiotic bacteria (Figure 2). In addition, we must consider that genetic differences in the expression of host receptors, the variable composition of the autochthonous microbiota in different individuals, and other host factors that contribute to the response to bacterial signals are likely to explain the variability in responses to probiotics in responding and non-responding individuals (Salonen et al., 2014).

## Immunomodulatory effectors

A significant number of relevant studies have highlighted the immunomodulatory effects that *Lactobacillus* and *Bifidobacterium* strains exert on the host immune system. For instance, there is evidence that *Bifidobacterium bifidum* LMG13195 and *Bifidobacterium breve* IPLA 20004 enhance intestinal barrier function and preferentially elicit Treg cell differentiation, which induces the expression of anti-inflammatory cytokines, when co-cultured with the human colorectal adenocarcinoma cell line HT29 (López et al., 2012). *Lactobacillus rhamnosus* GG interacts with macrophages in such a way that activated macrophages can discriminate between probiotic and pathogenic bacteria by INF-mediated TLR gene regulation (Miettinen et al., 2008), and the interaction between *Lactobacillus casei* CRL 431 and gut-associated immune cells induces an increase in the number of CD-206 and TLR2 receptors (Aragón et al., 2014).

The mediators of these interactions are largely unknown, although surface and cell-envelope molecules have been identified as some of the main players. Among them, we can distinguish between proteins and other components, such as peptidoglycan (PG), exopolysaccharides (EPS), teichoic acids (TA), and lipoteichoic acids (LTA). Known molecular effectors that mediate immunomodulatory mechanisms are listed in Table 1.

**Table 1:** **Examples of immunomodulatory effectors produced by classic/emerging probiotics**.

**Immunomodulatory effector**	**Species**	**Probiotic type**	**Effect on host immune system**	**References**
Surface Layer Protein A (SlpA)	*L. acidophilus*	Classic	Immunomodulation of intestinal dendritic cells	Konstantinov et al., 2008
Pili proteins (SpaCBA)	*L. rhamnosus*	Classic	Contact with mucosal cells	Reunanen et al., 2012
Pili	*B. bifidum*	Classic	Increase TNF-α and decrease IL-10 production	Turroni et al., 2013
	*B. breve*	Classic	Host colonization	O'Connell Motherway et al., 2011
Fimbriae	*E. coli*	Emerging	Host-colonization	Kleta et al., 2014
	*L. plantarum*	Classic	Immunomodulation	Murofushi et al., 2015
Serpin	*B. longum*	Classic	Human neutrophil and pancreatic elastase inhibitor	Ivanov et al., 2006
Serine-threonine rich proteins	*Bifidobacterium sp*.	Classic	Intestinal homeostasis	Nezametdinova et al., 2014
	*Lactobacillus sp*.	Classic	Intestinal homeostasis	Zakharevich et al., 2012
Serine-threonine rich peptide (STp)	*L. plantarum*	Classic	Anti-inflammatory; modulates intestinal dendritic cell function	Bernardo et al., 2012; Al-Hassi et al., 2014
Lactocepin	*L. paracasei*	Classic	Hydrolyzes IP-10	von Schillde et al., 2012
Secreted 15 kDa protein	*F. prausnitzii*	Emerging	Anti-inflammatory	Quévrain et al., 2015
Exopolysaccharides	*B. breve*	Classic	Immunomodulation	Fanning et al., 2012
	B. lactis^*^	Classic	Immunomodulation	Hidalgo-Cantabrana et al., 2014
Unmethylated CpG DNA	*Bifidobacterium sp*.	Classic	Induces Th1 response	Ménard et al., 2010
Teichoic/Lipoteichoic acids	*L. plantarum*	Classic	Anti-inflammatory	Grangette et al., 2005
Butyrate	*R. hominis F. prausnitzii A. muciniphila*	Emerging	Anti-inflammatory	Maslowski et al., 2009

* *Synonym of B. animalis subsp. lactis*.

### Surface proteins

Cell surface proteins include the S-layer proteins (Slp), which constitute the major surface proteins of some lactobacilli. In *Lactobacillus helveticus* fb213, *Lactobacillus acidophilus* fb116, and *L. acidophilus* fb214, Slp are well studied, and it is likely that they are necessary for lactobacilli survival in the gastrointestinal tract, as they can bind to components of the extracellular matrix, such as collagen and fibronectin, of intestinal cells (Meng et al., 2014; Yadav et al., 2015). Konstantinov and colleagues used an *slpA* knockout mutant of *L. acidophilus* to show that the interaction occurs via the recognition of SlpA by a specific receptor of dendritic cells, denominated DC-SIGN (Konstantinov et al., 2008). Additionally, proteins from structures that are included in the PG layer, such as pili, fimbria, and flagella, are recognized by the host immune system. Recently, it has been reported that bacterial SpaCBA pilus fibers in *L. rhamnosus* GG may be responsible for its well-known adhesion properties and confer the ability to contact host cells (Reunanen et al., 2012). *B. bifidum* PRL2010 pili have been shown to induce TNF-α production and decrease IL-10 production in the mouse mucosa, as well as to adhere to diverse extracellular matrix proteins (Turroni et al., 2013), while *B. breve* UCC2003 pili are essential for host colonization (O'Connell Motherway et al., 2011). In another recent work, gene complementation studies were used to show that the fimbriae of the probiotic strain *E. coli* Nissle 1917 were involved in the adhesion to porcine intestinal cells, thereby helping to prevent infection with enteropathogenic *E. coli* (EPEC) (Kleta et al., 2014).

### Cell wall non-proteinaceous components

Non-proteinaceous cell wall components have different roles in microbe-host crosstalk. It has been shown that the EPS from *Lactobacillus* and *Bifidobacterium* strains can have a modulator role in preventing pathogen invasion, even though the EPS of pathogenic bacteria have been classically viewed as possible virulent factors. Examples of immunomodulatory EPS are those from *B. breve* and *Bifidobacterium animalis* subsp. *lactis* (Fanning et al., 2012; Hidalgo-Cantabrana et al., 2014) or *Lactobacillus plantarum* strains (Murofushi et al., 2015). TAs are linear polymers of ribitol phosphate or glycerol phosphate that are covalently bound to D-alanine, monosaccharides, or amino sugars, and they are attached either to PG (wall TAs) or to the cytoplasmic membrane (membrane TAs or lipoteichoic acids; LTAs). TAs from *L. plantarum* were shown to display anti-inflammatory properties, as shown by the different cytokine production profiles of peripheral blood mononuclear cells (PBMCs) and monocytes exposed to this molecule (Grangette et al., 2005). In addition, mice fed a diet supplemented with *L. plantarum* LTAs or with an LTA-producing strain showed better scores in a colitis model compared with the control group and mice that were fed a *L. plantarum* LTA-deficient strain (Grangette et al., 2005). Although there have been a few promising results, this topic requires further research to clarify the mechanisms of action of the cell wall components of probiotics on the human gut microbiota.

### Soluble compounds

Soluble components that are produced by probiotic bacteria can also affect the bacterial-host interplay. In *Bifidobacterium longum* the secretion of serpin, a serine protease inhibitor, which specifically binds and inactivates human neutrophil and pancreatic elastase, was shown to contribute to gut homeostasis (Ivanov et al., 2006). Additionally, it has been observed that some proteins with characteristic biochemical motifs that are produced by both commensal and pathogenic bacteria can elicit specific functions and affect immune cells of the intestinal lumen. This is the case for a family of serine-threonine rich proteins, which was described in species of *Lactobacillus* and *Bifidobacterium*, with a recently described kinase function (Zakharevich et al., 2012; Nezametdinova et al., 2014). In lactobacilli, a serine-threonine peptide, STp, which is contained in a protein secreted by *L. plantarum*, was shown to be involved in bacterial aggregation (Hevia et al., 2013). Additionally, this peptide can modulate the dendritic cell phenotype of ulcerative colitis (UC) patients (Bernardo et al., 2012; Al-Hassi et al., 2014). It was also demonstrated that the immunomodulatory effect of *Lactobacillus paracasei* is mediated, at least in part, by the secreted protease lactocepin, which selectively degrades the chemokine IFN-γ-inducible protein 10 (IP-10) that functions in lymphocyte recruitment (von Schillde et al., 2012). There are other examples of non-proteinaceous compounds that can exert certain effects on the host. Some species of *Bifidobacterium* possess unmethylated CpG motifs in their DNA that were able to induce TLR9 activation, which is known to trigger a Th1 orientation of the immune system (Ménard et al., 2010). In contrast, in other studies, it was shown that intragastric and subcutaneous administration of DNA from a probiotic mix ameliorated the severity of colitis in a murine experimental colitis model, whereas a methylated probiotic DNA had no effect (Rachmilewitz et al., 2004).

## Emerging probiotics, a novel source of immunomodulatory effectors

In addition to *Lactobacillus* and *Bifidobacterium*, other microorganisms have received substantial interest among researchers as potentially new, beneficial gut bacteria. Most of them are common colonizers of the human gut under normal conditions. Some of these microbial types are considered to be markers of dysbiosis in intestinal inflammatory diseases, such as UC and Crohn's disease (Manichanh et al., 2006; El Aidy et al., 2013). In these conditions, a loss of microbial diversity and a significant reduction of members of *Clostridium* clusters IV and XIVa have been reported, particularly in bacteria involved in butyrate and propionate metabolism, such as *Ruminococcus, Eubacterium, Roseburia*, and *Faecalibacterium*. In this section, we will highlight current research on *F. prausnitzii* and *A. muciniphila*, two bacteria that have received much attention during the last few years because of their potential immunomodulatory properties.

*F. prausnitzii* is a “novel” intestinal bacterium whose immunomodulatory properties have been well characterized *in vitro* and *in vivo*. This anaerobic, Gram-positive bacterium seems to play a role in the maintenance of gut homeostasis, and its population is normally reduced in intestinal inflammatory diseases (Sokol et al., 2008; Cao et al., 2014; Machiels et al., 2014). In 2008, Sokol and colleagues studied the effects of whole bacteria, a cell culture supernatant, bacterial DNA, or membrane-derived fractions *in vitro* using the Caco-2 epithelial colorectal adenocarcinoma cell line and PBMCs, as well as *in vivo* in a mouse model of 2,4,6-trinitrobenzene sulfonic acid (TNBS)-induced colitis (Sokol et al., 2008). The results showed that *F. prausnitzii* cells exerted anti-inflammatory effects in PBMCs. Furthermore, its culture supernatant reduced IL-8 secretion and abolished the activation of nuclear factor kappa-light-chain-enhancer of activated B cells (NF-κB) in Caco-2 cells. Moreover, although no significant improvement of the disease was detected in mice, partial disease scores significantly improved in colitic mice receiving the cell culture supernatants, compared with the non-receiving group. Accordingly, the authors hypothesized that the beneficial effects of *F. prausnitzii* must be executed by a soluble compound that is secreted by the bacteria. In relation to this, recent work showed that *F. prausnitzii* secreted a 15-kDa protein with anti-inflammatory properties. This protein was able to inhibit the NF-κB pathway in intestinal epithelial cells, and it prevented colitis in an animal model (Quévrain et al., 2015). Additional research showed that this bacterium restored physiological parameters and downregulated cytokine profiles in mice with colitis, as well as increased the Treg population to a greater degree than other commensals such as *B. longum* (Qiu et al., 2013; Martín et al., 2015). UC patients have fewer butyrate-producing *Roseburia hominis* and *F. prausnitzii* (Machiels et al., 2014). It is likely that a significant part of their anti-inflammatory action results from the effect of SCFAs in colonocytes, as acetate, propionate, and butyrate modulate the inflammatory responses of immune cells through receptors such as Gpr43 and Gpr41 (Maslowski et al., 2009). However, despite all the information that has recently been discovered about these bacterial groups in healthy and diseased states, and besides butyrate seeming to be the key homeostasis promoter, additional work is required to elucidate the molecular mechanisms through which *F. prausnitzii* interacts with the host gut environment.

*A. muciniphila* is another common member of the healthy gut microbiota in humans at all stages of age (Collado et al., 2007; Belzer and de Vos, 2012). *A. muciniphila* is a Gram-negative, strictly anaerobic, mucin-degrading microorganism member of the *Verrucomicrobia* phylum, and it was one of the first bacteria shown to utilize mucin, the glycosylated protein layer that covers the gut epithelium, as its sole carbon, nitrogen, and sulfur source. The products derived from mucin degradation are mainly SCFAs that feed colonocyte metabolism and confer health properties to the host. By degrading the mucin of the external mucus layer, *A. muciniphila* helps with the continuous renovation of the protective cover of the mucosae, and it maintains a healthy protective barrier that prevents the entrance of enteropathogens into the epithelium (Lukovac et al., 2014). In addition, when *A. muciniphila* was administered to mice, there were increased intestinal levels of endocannabinoids that control inflammation, the gut barrier, and gut peptide secretion, suggesting an immunomodulatory role for this bacterium (Everard et al., 2013).

## Concluding remarks

In conclusion, even though much effort has been put into probiotic research during recent decades, the mechanisms underlying the immunomodulatory effects of beneficial intestinal bacteria have scarcely been elucidated. There is compelling evidence that novel bacterial players, other than *Lactobacillus* and *Bifidobacterium*, could play a role in these processes and are much more important than previously thought; however, difficulties in growing some of these bacteria on laboratory- and industrial-scales, and the lack of molecular tools needed to perform functional genomic analyses, seriously hamper the characterization of novel strains. Further research is needed to overcome these culturing and functional characterization difficulties to perform well-designed pre-clinical and intervention studies that shed new light on the mechanisms responsible for the beneficial effects attributed to these bacteria.

## Author contributions

AH, SD, BS, and AM contributed to the conception and design of the work, and to the acquisition, analysis, and interpretation of the data. All authors contributed to the drafting of the manuscript and approved the final version to be published.

### Conflict of interest statement

The authors declare that the research was conducted in the absence of any commercial or financial relationships that could be construed as a potential conflict of interest.
